# A Non-Stressful Temperature Rise and Greater Food Availability Could Increase Tolerance to Calcium Limitation of *Daphnia* cf. *pulex* (Sensu Hebert, 1995) Populations in Cold Soft-Water Lakes

**DOI:** 10.3390/biology11101539

**Published:** 2022-10-20

**Authors:** Eloísa Ramos-Rodríguez, Carmen Pérez-Martínez, José María Conde-Porcuna

**Affiliations:** 1 Departamento de Ecología, Facultad de Ciencias, Universidad de Granada, 18071 Granada, Spain; 2 Instituto del Agua, Universidad de Granada, 18003 Granada, Spain

**Keywords:** calcium limitation, cladocera, *Daphnia pulex*, food quantity, warming

## Abstract

**Simple Summary:**

Climate warming is evident at many cold sites, including high-altitude and high-latitude lakes. The cladoceran *Daphnia pulex*, with high calcium (Ca) demands for its heavily calcified exoskeleton, is an ecologically important zooplankton species inhabiting these lakes, many of which are Ca-limited. Other stressors, such as warming and food limitation, may interact with Ca limitation and affect *Daphnia* populations adapted to these cold environments. In a study of factorial design, a clone of North American *Daphnia* cf. *pulex* was exposed to a low-Ca gradient, to temperature rises within its preferred range and expected in these lakes, and to conditions of low and high food quantity. Results suggest that climate warming and higher food availability will make *D. pulex* populations more tolerant to Ca limitation. Changes in the abundance of *Daphnia* may be relevant to ecological processes and the functioning of aquatic ecosystems.

**Abstract:**

Calcium (Ca) is an important driver of community structure in freshwaters. We examined the combined effects of increased temperatures and variations in food quantity on the tolerance to low Ca of *Daphnia pulex*. The aim was to predict the impact of climate warming on this keystone zooplanktonic species in cold-climate lakes. We conducted a factorial life-history experiment in a clone of North American *Daphnia* cf. *pulex* to analyse the interaction effects of a temperature increase (17.5 °C–21 °C) within their physiological preferred range and expected by climate warming over the next few decades and a narrow Ca gradient (0.25–1.74 mg Ca L^−1^) under stressful vs. abundant food conditions. We found a striking positive synergistic effect of Ca and temperature on *D. pulex* reproduction at high food conditions. Although the increase in temperature to 21 °C greatly reduced survival, high energy allocation to reproduction at high food levels allowed the population to succeed in poor Ca (<0.25 mg Ca L^−1^). Results suggest that climate warming and higher food availability will make the populations of many cold and Ca-limited lakes more tolerant to low Ca levels with higher growth population rates, thereby altering zooplanktonic community structures and inducing potential cascading effects on the food web.

## 1. Introduction

Organisms and populations must cope with multiple simultaneous stressors that affect their biological fitness and threaten their persistence [1]. A crucial aim of evolutionary ecological research and conservation is to evaluate the potential interactive effects of these stressors on life history traits in order to predict the adaptation and evolution of organisms in affected ecosystems [2,3]. However, investigation of the complex interactions between ecological factors on natural populations is hampered by the inherent complexity of ecosystems [4]. In this context, factorial laboratory studies under multiple stressors are essential to make some predictions about when and where different types of ecological factors may interact.

*Daphnia* (O.F. Müller 1785), a very common planktonic grazer in lakes and ponds, is a model genus in evolutionary and ecological functional genomics [5]. *Daphnia* plays a key role in aquatic food webs because of its trophic position, linking phytoplankton with higher trophic levels of invertebrate and fish predators. They are also considered sentinel species of freshwater environments [6], where changes in their population abundance have been associated with warming, food quantity and calcium (Ca) limitation, among other environmental stressors. The ecology and evolution of *Daphnia* are well documented under conditions of increasing temperature and changing algal biomass. However, there has been less research on the potential interactions of water Ca-limitation with warming [7,8] and food quantity [7,9]. This is of critical ecological concern because of the evident climate warming in many regions of the world that contain Ca-limited lakes with varying trophic states. The Ca demands of freshwater daphniids are elevated because of their heavily calcified exoskeleton and regular moult cycle [10], with neonates and juveniles being less tolerant to low Ca concentration than adults due to their rapid growth [11,12]. The *D. pulex-pulicaria* complex is one of the most vulnerable *Daphnia* species to Ca limitation because of its high Ca needs [8,13]. Studies have also reported combined effects on daphniid survival and reproduction of Ca stress and its potential interactions with UV radiation [14], metal and pharmaceutical toxicity [15,16,17], poor food quality [8,18,19,20], and the presence of invertebrate predators [21]. The swimming behaviour of *Daphnia* may also be influenced by low Ca availability at higher temperatures [22]. Finally, Huang et al. [23] recently reported that a decline in Ca reduced the numbers of ephippia and resting eggs of *D. pulex*, further endangering the persistence of this keystone species.

A recent global analysis of Ca in freshwater systems identified numerous large lakes and reservoirs with Ca < 1.5 mg Ca L^−1^ [24], considered a limiting concentration for *Daphnia* reproduction. Lakewater Ca has declined in boreal regions due to the accelerated leaching of Ca from soils linked to high acidic deposition (e.g., [25]), among other causes. However, although anthropogenic acid deposition has been reduced, a global decline in freshwater Ca continues towards pre-industrial levels, a somewhat paradoxical response [24,26]. By contrast, increased Ca has been documented in many high-mountain Mediterranean ecosystems such as Sierra Nevada (Southeast Spain) and the French and Italian Alps due to greater atmospheric Ca input from Saharan dust deposition [27,28,29]. An increase in the frequency and intensity of Saharan dust events in Central Europe over the past decade has been associated with climate change (e.g., [30]), and the Copernicus Atmosphere Monitoring Service (CAMS) described recurrent large plumes of Saharan dust moving north across southern and central Europe in 2022 (https://atmosphere.copernicus.eu/ (accessed on 20 September 2022). Records for peak concentrations in Southern Spain were broken in March 2022, with the plume reaching as far as Scandinavia over the next few days.

Temperature is a key factor in the life cycle and fitness of all aquatic ectotherms, and its effects on the metabolic rate, survival, development time, reproduction, filtration capacity and body size of *Daphnia* are well documented [31,32,33]. However, the degree of impact on biological processes depends on the range of temperatures to which an organism is exposed, among other variables [33,34]. According to Shelford’s species tolerance curves, metabolic and growth rates of species increase with higher temperature within the preferred temperature range of the species (i.e., optimal physiological temperature or central zone of the curve) [35]. At temperatures above the preferred range, ectotherms can survive but their metabolic and growth rates decrease [36,37]. Daphniids show wide inter- and intraspecific variability in their temperature tolerance ranges [38,39] and in the thermal optimum for their development and reproduction [31]. For example, 16–21 °C has been proposed as the preferred range for species of the *D. pulex-pulicaria* complex, with maximum offspring production [31,39,40,41], whereas some populations have a thermal optimum as high as 24 °C [7]. Differences in thermal tolerance among *Daphnia* species and between clones have been related to the evolutionary history of genotypes [38,42]. The thermal tolerance of *Daphnia* is also known to be strongly dependent on the acclimation temperature, increasing at higher temperatures [38,43].

Climate warming, a key feature of the Anthropocene [44], is particularly evident in cold-climate ecosystems such as high-mountain lakes worldwide [45,46,47] and high-latitude lakes in America and Europe [48,49]. Mountain regions have experienced some of the highest recorded rates of air warming [46,50], with an average annual increase of up to 0.6 °C decade^−1^ [47]. Increases in maximum surface water temperature observed in some European high-latitude lakes have been associated with variations in air temperature and the greater frequency of warm extremes, lengthening periods in which critical temperatures are exceeded (>20 °C) (see [51]). Hence, aquatic species such as the *Daphnia* genus could be subjected to both Ca limitation and temperature rises in many cold lakes over the coming decades. In this way, changes in daphniid species have been reported in some soft-water alpine lakes in Sierra Nevada over the past century, in part associated with increased water Ca from Saharan Ca deposition and warming [29,52,53], whereas a decline in *Daphnia* populations has been observed in North America boreal lakes due to Ca limitation from acid deposition [13]. *Daphnia pulex-pulicaria complex* is one of the most common planktonic cladocerans in these cold Ca-limited lakes [13,53,54], and changes in its abundance are likely to result from the expected warming in these regions, inducing potential top-down and/or bottom-up effects of *D. pulex* on the food web. However, the Ca in these soft lakes is also changing due to various factors, making it difficult to predict the result of temperature × Ca interaction. We have only traced one study that simultaneously tested the combined effects of Ca deficiency, increased temperature (20–32 °C) and algal resource availability on life-history traits of daphniids [7]. To date, however, the effect of Ca limitation on *Daphnia* populations has not been examined within their preferred temperature range (16–21 °C for *D. pulex*), which will likely be maintained in most high-altitude and high-latitude lakes over the next few decades [47,51].

The aim of this study was to explore the effect on *D. pulex* populations of Ca limitation in cold-climate lakes with varying algal resource availability when the temperature is kept within their preferred range and the temperatures expected due to climate warming. For this purpose, North American *Daphnia* cf. *pulex* (sensu Hebert, 1995) (hereafter *D*. *pulex*) were collected from a soft-water lake in Sierra Nevada Mountains (Spain) for a life-history experiment to evaluate the combined effects of a non-stressful temperature increase (17.5–21 °C) and a narrow water Ca gradient (0.25–1.74 mg Ca L^−1^) under stressful *versus* abundant food conditions. This *Daphnia* lineage is widely distributed in every continent except Antarctica [55]. Effects were determined at individual (survival and development and reproduction traits) and population (intrinsic rate of natural increase) levels. The experimental Ca values are within the natural range recorded in some alpine lakes of Sierra Nevada with the presence of European *D. pulicaria* and North American *D. pulex* lineages [53,54,56]. Peaks in maximum surface temperature of around 20–21 °C have been observed in some shallow Sierra Nevada lakes but are usually 17–18 °C in most lakes containing *D. pulex-pulicaria* ([56], personal observation), and the frequency of peaks of 18 °C has increased during recent dry and hot years. Ca limitation and warming are recognised as environmental stressors for crustacean zooplankton communities in many soft-water lakes with varying trophic states and often dominated by the Ca-rich *Daphnia.* The objectives of the present study were: (i) to determine the response of *D. pulex* populations under Ca limitation to temperature increases within their preferred range and expected due to climate warming; (ii) to determine whether Ca thresholds for survival and reproduction differ for *D. pulex* living at lower temperatures in many other cooler lakes; and (iii) to determine whether Ca thresholds are influenced by food availability.

## 2. Materials and Methods

### 2.1. Collection Site

**Figure 1:**
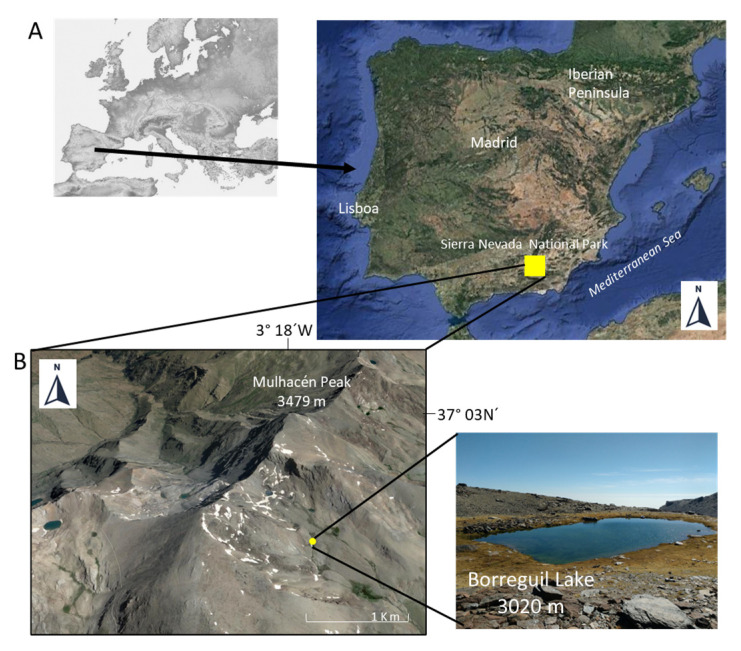
(**A**) Map of Europe and map of the Iberian Peninsula showing the location of the study area. (**B**) Geographic location of Borreguil Lake in Sierra Nevada National Park (Spain). Source Google Earth Pro, image: Landsat/Copernicus, imagery data 14 December 2015. Borreguil Lake photograph (E. Corral-Arredondo, 25 September 2017) taken from https://lagunasdesierranevada.es/ (accessed on 10 October 2022).

For this study, a *Daphnia pulex* clone (termed BG17) was originally isolated from a plankton sample from Borreguil (BG) Lake (37°03′ N, 3°17′ W) in Sierra Nevada (Figure 1). The sample was collected with a conical plankton net (40 µm mesh) in the summer of 2017, and the water temperature at the time of collection was 11.7 °C. In the Sierra Nevada mountains, ~50 small lakes of glacial origin lie at an elevation of ~2800–3100 m a.s.l., all on siliceous bedrock. These high-mountain lakes have typically a surface area < 1 ha and they are shallow (maximum depth < 10 m), fishless, and with low primary production. Further details can be found in [52,53], among others. In particular, BG Lake (Figure 1B) is a small (0.18 ha) and permanent shallow lake (maximum depth ≈ 2.5 m), with low values of alkalinity (range: 0.05–0.09 meq L^−1^), conductivity (range:11.00–13.63 µS cm^−1^) and primary production (chlorophyll-a range: 0.25–4.3 µg L^−1^) throughout the ice-free period; DOC values range from 0.6–1.58 mg L^−1^, pH values from 6.25–8.3, total phosphorous values from 2.5–26.33 µg L^−1^ and total nitrogen values from 180–2196 µg L^−1^ ([52,53,54], Villar-Argáiz (unpublished data, from July 2020), Pérez-Martínez (unpublished data, from September 2021)). This lake has low Ca (mean: 1.03 ± 0.14 (SD), range: 0.8–1.2 mg Ca L^−1^ [53,54], which may be limiting for the reproduction of cladoceran taxa such as *D. pulex-pulicaria complex* [7,9,21]. Gutiérrez-Parejo [57] recorded an average and maximum abundance of *D. pulex* of 4.23 ± 6.72 (SD) (*n* = 3) and 12 individuals L^−1^ in the summer of 2017, respectively.

### 2.2. Culturing Daphnia and Algae

**Figure 2:**
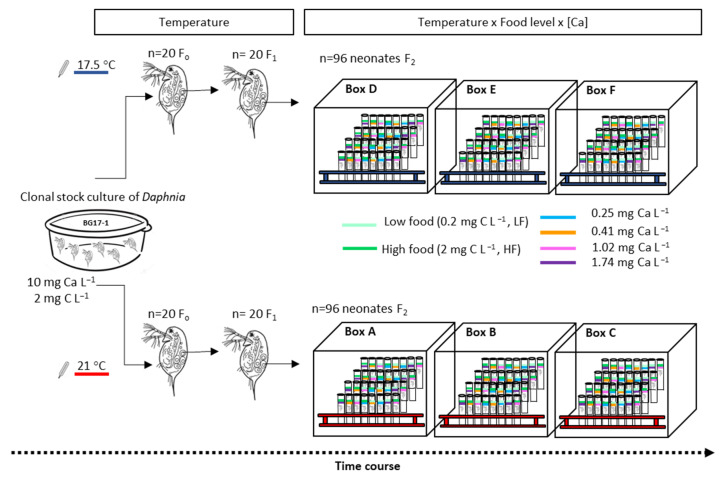
Schematic diagram of the experimental design to test the combined effects of a Ca gradient at two temperatures and low (LF) or high (HF) food quantity on the survival and reproduction of *D. pulex*.

Uniclonal stock cultures of *D*. *pulex* were maintained for two years at the Institute of Water Research at the University of Granada with soft COMBO medium [58] in 0.5–2 L glass vessels in an isolated room (6 m^2^) at a controlled temperature of 17.5 ± 0.7 °C, with ~100 µmol quanta m^−2^ s^−1^ photosynthetically active radiation (PAR) on a 15 h:9 h light:dark cycle (Figure 2). *Daphnia* were fed ~7.5 × 10^4^ cells ml^−1^ of *Scenedesmus* sp. (~2 mg C L^−1^) three times a week (Monday, Wednesday, and Friday), and 25% of the COMBO medium was renewed every week; this medium has a Ca content of 10 mg L^−1^ [58]. Genus *Scenedesmus* cells have proven to be a good food for *D. pulex* [59]. A preliminary growth experiment determined that 1 mg C L^−1^ is sufficient to support a moderate population growth rate of the *D. pulex* clone (0.152 ± 0.002 (standard error [SE]) d^−1^, *n* = 10) fed the same *Scenedesmus* strain and maintained at 17.5 ± 0.7 °C. The carbon level used for maintaining daphniids is higher than the upper threshold of 0.7 mg C L^−1^ for egg production of *D. pulex*, where the curve reaches a plateau determined by Lampert [59].

The chlorophyte *Scenedesmus* sp. (length 13.77 ± 2.09 µm [mean ± SD], width 5.23 ± 1.53 µm, biovolume 197 µm^3^, *n* = 20) was also from the culture collection at the Granada University Institute of Water Research. This strain of *Scenedesmus* (designated IdA Sce/RS) was also originally isolated from another lake in Sierra Nevada (Río Seco Lake) and has been maintained for 10 years in exponentially growing semicontinuous batch cultures with COMBO medium [58]. *Scenedesmus* was routinely cultured in 0.6 L volume in the same isolated room at 17.5 ± 0.7 °C with sterile-filtered air and ~100 µmol quanta m^−2^ s^−1^ PAR on a 15 h:9 h light:dark cycle. Exponential growth was maintained by harvesting 50% of the volume of the culture weekly and replacing it with a new COMBO medium to bring the culture to the original volume [60]. Harvested algae were stored in a refrigerator at 4 °C until used to feed *Daphnia* cultures. Algae stored longer than one week were discarded. Although the *Scenedesmus* culture was not axenic, it was unialgal and free of protozoan and fungal contaminants, and sterile techniques were always used. Under these culture conditions, the per-cell carbon content of *Scenedesmus* was 30.87 ± 0.52 pg (mean ± SD, *n* = 20), estimating the carbon content from cell volume measurements by regression from Menden-Deuer & Lessard [61].

For this experiment, the harvested algae in log-phase growth were centrifuged (3000 rpm for 5 min) every two or three days and resuspended in Ca-free COMBO medium to prevent the addition of Ca to experimental *Daphnia* individuals. The use of algae harvested in the log-phase of growth minimises possible effects due to food quality variation because phytoplankton consume nutrients at an optimal ratio while nutrients are high [62]. Cell density in the algal suspension was estimated with an improved Neubauer hemacytometer.

### 2.3. Experimental Design

A life table experiment with a 4 × 2 × 2 factorial design was undertaken to explore how the combined effects of aqueous Ca concentration, food quantity and temperature affect the growth, reproduction and survival of *Daphnia* (Figure 2). Ca treatments were obtained by adjusting the concentrations of CaCl_2_·2H_2_O in the COMBO medium recipe.

Each nominal Ca treatment (0.25, 0.5, 1.0 and 2.0 mg Ca L^−1^) was combined with two food levels (low [0.2 mg C L^−1^, LF] and high [2.0 mg C L^−1^, HF]), and two temperatures (17.5 and 21 °C). The purpose of these food levels was to create stressful vs. abundant food conditions for *D. pulex* reproduction according to the life table studies of Pérez-Fuentetaja & Goodberry [9]. It can be expected that the carbon biomass in HF treatments is sufficient for optimal reproduction and that adults will survive and reproduce with an intrinsic rate of increase in the population >0 in LF treatments, although with a lower rate than in HF treatment [9]. Ca levels initially contemplated in our experimental design were selected to induce various intensities of Ca limitation stress in the experimental animals, being within the natural range of this element in BG Lake and other alpine lakes of Sierra Nevada (0.3–5.6 mg Ca L^−1^) [9,54]. The Ca concentration of each medium was tested (before addition of food) at the start of the experiment and weekly to test for potential changes in the actual Ca concentration in the COMBO medium due to EDTA chelation effects [16]. Ca samples were analysed at the Centre for Scientific Instrumentation at the University of Granada with an Optima 8300 ICP-OES spectrometer from Perkin-Elmer. The Ca measured in the *Daphnia* growth medium matched well with the planned Ca, establishing that the mean (±SD) experimental [Ca] values were 0.25 ± 0.05, 0.41 ± 0.08, 1.02 ± 0.19 and 1.74 ± 0.10 mg Ca L^−1^, respectively.

The experiment was performed in the same room as the *Daphnia* stock cultures for the temperature of 17.5 °C and in a Memmert incubator (ICP 600) for the temperature of 21 °C. Minimum and maximum temperatures were recorded during experiments using a minimum/maximum thermometer, finding the mean (±SD) experimental temperatures to be 17.5 ± 0.7 and 20.8 ± 0.7 °C. In order to independently analyse the effect of temperature increase and avoid pseudoreplication [63], the experimental units were randomly placed in six wooden boxes of 30 cm^3^ (three at each temperature, Figure 2). The boxes were opaque and provided with a Zenit LED light with a PAR irradiance of 90–120 µmol quanta m^−2^ s^−1^ and a 15 h:9 h light:dark photoperiod. This light intensity does not change the temperature of the chambers [64].

In order to exclude maternal effects in life history experiments and minimise variance among test animals, all experimental individuals should preferentially be cultured under treatment conditions for at least two generations and taken from the third clutch before trials take place [65]. However, as the parental generation in our experiment experienced low reproduction in 0.2 mg C L^−1^ (see Results section), achievement of a sufficient number of experimental neonates required that both food concentration treatments contained F2-generation neonates (<24 h old) from only the first brood of *Daphnia* growing in original COMBO at high food concentration (2 mg C L^−1^). In this way, before the start of the experimental life-history trials, 20 females (F0) with mature eggs were isolated at each temperature from stock cultures into 250-mL glass beakers containing COMBO (10 mg Ca L^−1^) and algae (2 mg C L^−1^) until their offspring were released. The neonates (F1) were then isolated individually in 25 mL glass vials filled with 20 mL of original COMBO medium and algae (2 mg C L^−1^). Food and culture media were replaced every second day to clean vials, and neonates (F2) of the first brood of these *Daphnia* (already acclimated to the experimental temperature) were used for the experiments. Because the age at first reproduction of F1-females varied between temperatures (see the Results section), the starting day of the experiment differed among experimental units. Offspring born from each F1-mother over a period of five days were taken and distributed randomly among Ca and food treatments. The first clutches ranged from four to seven neonates at 17.5 °C and from four to nine neonates at 21 °C, and the variance in neonate body size between the two temperatures would be similar [66]. Moreover, experimental individuals were taken from mothers within a narrow age range to control the fitness of their offspring [65]. Twelve replicates per treatment were separated into individual vials, yielding 192 experimental units. All vials were sealed using parafilm to avoid evaporation, and 32-vial sets were randomly distributed in each wooden box in test-tube racks. Before trials started, it was confirmed that all experimental neonates were female. The experimental animals were placed in a clean vial and given fresh food and media every second day.

Finally, the oxygen concentration, conductivity, and pH of each Ca treatment (after addition of food) were recorded on day 0 of the experiment and then weekly using a YSI multiprobe system after pH calibration. All experimental media were oxygen-saturated (>90%) during the experiment. Mean conductivity and mean pH values ranged from 199.43 to 219.97 µS cm^−1^ and from 7.95 to 8.21, respectively. One-way ANOVAs showed no significant differences between Ca treatments (oxygen: F(3, 8) = 0.35, *p* > 0.05; conductivity: F(3, 8) = 0.17, *p* > 0.05; pH: F(3, 8) = 2.83, *p* > 0.05). Hence, manipulation of the Ca of the medium produced negligible changes in osmoregulation ability.

#### Quantified Life History Parameters

Data were collected daily for up to 19 d to quantify the following life history traits for each experimental individual: survival, age at maturity, average brood size (average number of neonates born in each clutch, i.e., fertility), reproduction output (i.e., number of total offspring) and number of moults. An endpoint of 19 d is adequate to determine the lethality and the age at first reproduction of *Daphnia pulex-pulicaria* under low Ca and low food conditions at temperatures of around 20 °C ([9], this study). Neonates were removed and discarded from trials. The age at which the individual produced the first egg(s) was the criterion for maturity. The moulting frequency of juveniles was calculated as the total number of accumulated moults up to maturity divided by the number of days taken to reach maturity. At the end of the experiment, body size (length of surviving adults) was also recorded, measuring the length of *Daphnia* individuals from the top of the head to the base of the tail spine.

Survivorship and reproductive data from abbreviated life tables were used to calculate the intrinsic rate of natural increase, *r.* The *r* is a common measure of the short-term population fitness in cycles with diapause investment, as observed in cladocerans [67], and it has been widely used to test the effect of different environmental factors on the performance of zooplankton species or clonal lines (e.g., [3,21,68]). It was estimated in the present study by solving the Euler–Lotka equation iteratively, assuming exponential growth:

(1) $$\sum e^{-r\left(x+0.5\right)}\cdot l_{x}\cdot m_{x}=1$$

where *e* is the Euler constant, *x* the age in days, *l_x_* the age-specific survival rate (i.e., the proportion of surviving individuals at day *x* relative to the initial number of females), and *m_x_* the age-specific fertility rate (i.e., the mean number of offspring produced on day *x* by a female of age *x*). The estimate of *r* was refined until the value of the left-hand side of Equation (1) was 10,000. When no offspring was produced in a treatment, the following formula was used to calculate the average *r*:

*r* = (ln(N_t_ + 0.01)/ln(N_0_ + 0.01))/t (2)

where N_t_ is the number of animals at the end of the experimental treatment, N_0_ the number of animals at day 0, and t the duration of the experiment (19 days). The constant value of 0.01 was added to N to make the calculation feasible (see [69]). Means and standard errors of these population demographic parameters were estimated by the jack-knife technique described in Meyer et al. [70], using the Microsoft Excel program (version 2013).

### 2.4. Statistical Analyses

R 3.4.3 (R Foundation for Statistical Computing) was mainly used for statistical analyses. A first analysis was conducted of the overall relationship of the hazard of *Daphnia* mortality with Ca, food concentration, temperature and their interactions. A discrete-time survival model with mixed effects was used [71], including the three predictor variables as fixed-effect continuous predictors and “box” (with six levels) as a random-effect factor. A Cox regression model could not be used due to violations of the assumption of proportional hazards for the predictors Ca and food. Unlike Cox regression models with mixed effects, discrete-time survival models do not require the assumption of a constant hazard function within each interval [71], and they can be applied when survival time is measured in discrete values (e.g., days to death) by using a discrete version of the hazard function [71]. To this end, the original survival data set was split into 19 intervals using the *survSplit* function of the *survival* package [72]. The resulting data served to develop a mixed model using the *glmmadmb* function of the *glmmADMB* package [73,74] from the R program, with a binomial distribution and a complementary log-log (*cloglog*) link function to model the probability that the death event occurred at a specified discrete time point on condition that it had not yet occurred [75]. Interaction terms were excluded from the model if statistical significance was not reached to check changes in the significance of main effects, given that some authors drop non-significant interactions for this purpose [76,77]. The random factor “box” was not influential in the model after the application of the likelihood ratio test (LRT), in which the log-likelihood was compared between the model and a reduced model (with identical fixed effect structure) from which the “box” effect was dropped [76].

The Ca survival threshold for *Daphnia* (i.e., lower lethal Ca concentration threshold) was considered according to Cairns & Yan [10] as the Ca concentration at which 50% mortality is observed over the experimental period and no reproduction is possible.

The combined impact of Ca concentration (log-transformed), food concentration and temperature on the age of maturity (log-transformed), the moulting rate of juveniles, the body size, the reproduction output (R_o_), the average brood size, and the population growth rate (*r*) were analysed using linear mixed-effects models (LMMs), in which the independent variables were included as fixed-effect continuous predictors and “box” as a random effect. These models were also constructed using the function *glmmadmb* in the *glmmADMB* R package [73,74]. Interaction terms were removed from the model if significance was not reached. The Kolmogorov–Smirnov normality test of standardised residuals, Bonferroni outlier test and Breusch–Pagan test for heteroscedasticity were applied to check model assumptions. However, R_o_ and *r* both violated the assumptions of normality and homoscedasticity; hence, their effects and those of their interactions were determined by performing null model permutation tests after 3000 random permutations in a linear mixed model of *lmer*, using the *permlmer* function in the *predictmeans* R package [78]. LRT application showed that the random factor “box” was not influential in any model related to these life-history traits [76].

The Ca reproductive saturation point for *Daphnia* was defined as the Ca concentration at which no significant difference in reproduction is observed in comparison to subsequent higher Ca concentrations [10].

Finally, a Ca concentration threshold for population growth was estimated to examine possible differences in the growth efficiency of *Daphnia* at low Ca between experimental temperatures, using the Monod model with a threshold for zero-growth to describe the relationship between *r* and Ca (mg Ca L^−1^), defined by Equation (3):

(3) $$r=r_{max}\frac{Ca-Ca_{ZPG}}{Ca-Ca_{ZPG}+Ks}$$

where *r_max_* is the maximal population growth rate, Ca_ZPG_ is the threshold [Ca] level for zero population growth, and K_s_ is the Monod constant. Fitting was computed using a non-linear fitting method that applies the Marquardt–Levenberg algorithm to determine the parameters (and their SEs). Only data obtained for HF treatments showed a good fit of the modified Monod equation with Ca level (regression *p*-value < 0.0001). Ca_ZPG_ could not be estimated for LF treatments because most *Daphnia* mean *r* values were negative. STATISTICA (v.7.1, StatSoft, Tulsa, OK, USA) was used for this analysis.

## 3. Results

### 3.1. Survival

**Figure 3:**
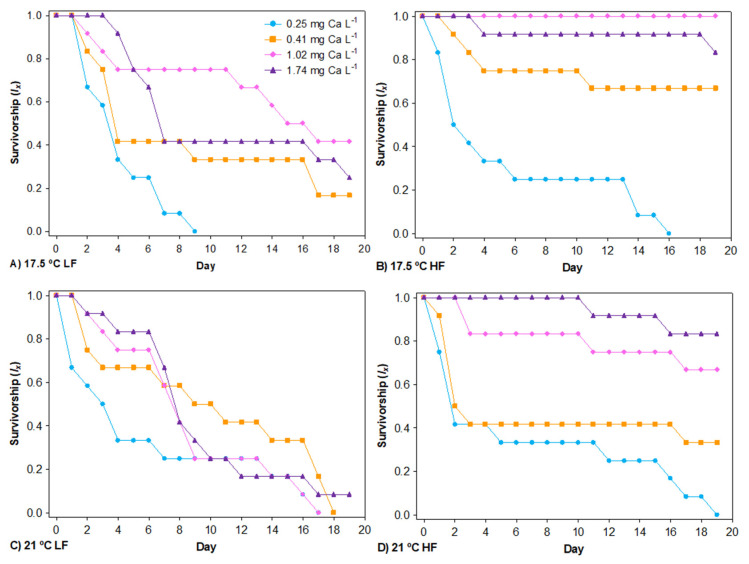
The age−specific survivorship (l_x_) of *D. pulex* when reared across a calcium gradient at two temperatures (17.5 °C and 21 °C) and two food levels (LF [low food] and HF [high food]) over a period of 19 days.

No daphniids survived for 19 d at 0.25 mg Ca L^−1^, regardless of the temperature or food concentration (Figure 3A–D). Neither Ca nor food concentration exerted significant single effects on the hazard of mortality. However, the interaction between Ca and food concentration affected survival (Table 1), and the hazard of mortality due to low Ca was higher with increased food quantity (Figure 3A–D). Temperature had a significant effect on survival, regardless of food concentration or Ca (Table 1 and Figure 3A–D), with a greater hazard of mortality of *Daphnia* at higher temperatures, observing a reduction in the average lifespan from 11.69 ± 0.80 (SE) days at 17.5 °C to 10.11 ± 0.75 (SE) days at 21 °C. Non-significant Ca x temperature and food concentration x temperature interaction terms were removed from the final model. Nonetheless, a survival rate >50% was observed at 0.41 mg Ca L^−1^ over 19 d at HF and 17.5 °C, (Figure 3B), when some daphniids reproduced. At HF and 21 °C, however, *Daphnia* had a mortality rate >50% at 0.41 mg Ca L^−1^ within the experimental timeframe (Figure 3D), and no animal reproduced at this Ca level. Hence, under a non-limiting food concentration, the Ca threshold of the *D. pulex* clone for surviving was between 0.25 and 0.41 mg Ca L^−1^ at 17.5 °C but between 0.41 and 1.02 mg Ca L^−1^ at 21 °C. However, all survival rates were <50% after 10 d at LF and the higher temperature (Figure 3C). Therefore, it was not possible to detect changes in the Ca survival threshold with temperature increase for the experimental Ca gradient at this food level.

### 3.2. Development Traits

#### 3.2.1. Age of Maturity

**Figure 4:**
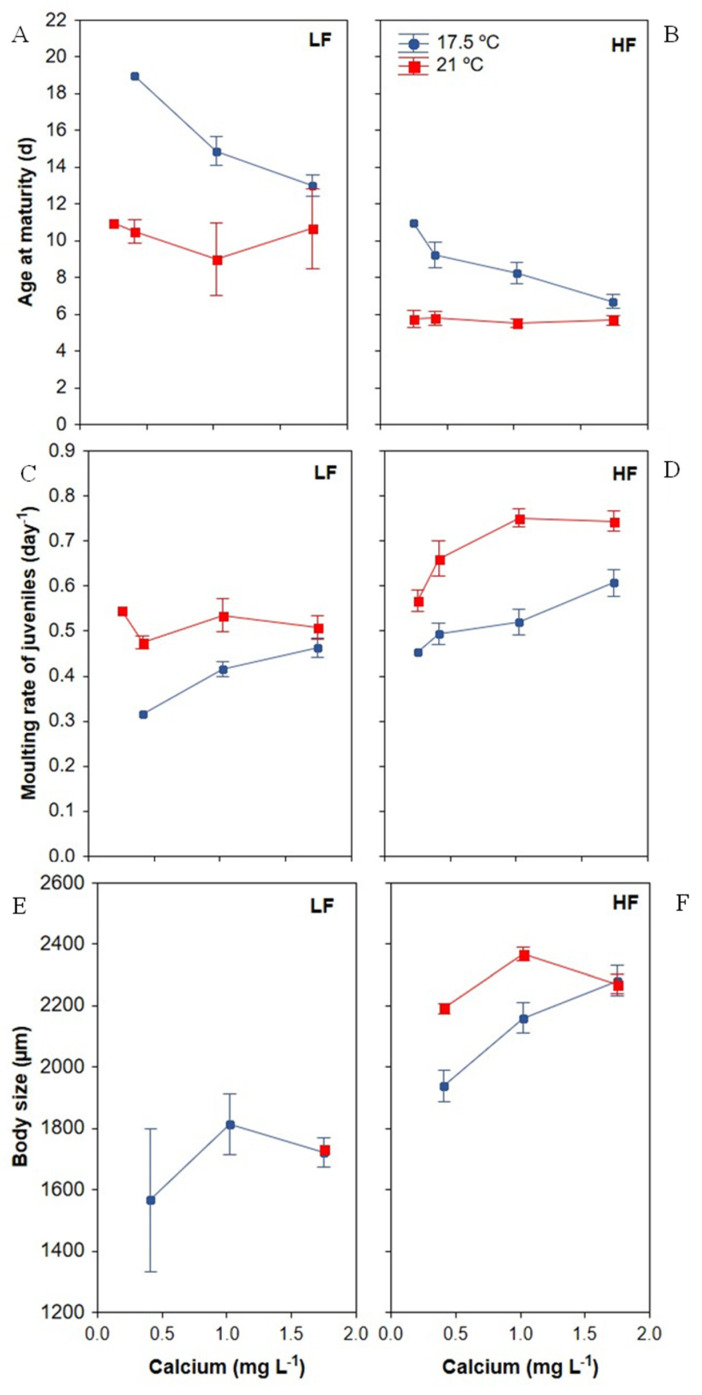
Effects of calcium concentration on development traits of *D. pulex* at two temperatures and low (LF) or high (HF) food level. Data represent mean ± SE. (**A**,**B**): Age of maturity. Error whiskers are not present for the 17.5 °C HF 0.25 mg Ca L^−1^ treatment because all individuals reproduced at the age of 11 days (*n* = 3); in the 17.5 °C LF 0.25 mg Ca L^−1^ treatment, no female reached reproductive maturity; in the 17.5 °C LF 0.41 mg Ca L^−1^ and 21 °C LF 0.25 mg Ca L^−1^ treatments, only one female reached reproductive maturity; (**C**,**D**): Moulting rate of juveniles. Data for the 17.5 °C LF 0.25 mg Ca L^−1^ treatment are absent because no female reached reproductive maturity; in 17.5 °C LF 0.41 mg Ca L^−1^ and 21 °C LF 0.25 mg Ca L^−1^ treatments only one female reached reproductive maturity; (**E**,**F**): Body size. Some data are not present because no animal survived on day 19.

Time to reproduction in *Daphnia* was significantly affected by the individual effects of food level, temperature and Ca (Table 2). Overall, higher food concentration, temperature and Ca advanced the age at maturity (Figure 4A,B). Among individuals grown at LF, reproductive maturity was reached by 24% and only attained before day 10 by 22% (Figure 4A); the remaining 76% died before reaching maturity. Age at maturity was greatly delayed at LF *versus* HF (12.57 ± 0.70 (SE) vs. 7.03 ± 0.26 (SE) days) and at 0.25 mg Ca L^−1^ *versus* 1.74 mg Ca L^−1^ (8.38 ± 1.02 (SE) vs. 7.36 ± 0.53 (SE) days). Regarding temperature, *Daphnia* maturity advanced from 9.98 ± 0.53 (SE) at 17.5 °C days to 6.83 ± 0.38 (SE) days at 21 °C. No significant interactions were found between Ca × food and food × temperature. However, there was a significant interaction effect between Ca and temperature (Table 2), finding an earlier age at maturity of *Daphnia* with increasing Ca at 17.5 °C, regardless of the food quantity. No effect of Ca gradient was observed at 21 °C (Figure 4A,B).

#### 3.2.2. Moulting Frequency of Juveniles

Overall, the number of moults during the juvenile phase ranged between four and seven moults in LF treatments and between three and six moults in HF treatments (Figure 4C,D). Juveniles experienced greater moulting frequencies up to the age of maturity when Ca and temperature increased. Moreover, an interaction between food concentration and temperature was also observed (Table 2) showing that the frequency of moulting during juvenile growth increased at the higher temperature and food level (Figure 4C,D).

#### 3.2.3. Body Size

The body size of *Daphnia* individuals that survived at day 19 was significantly larger at higher levels of food, temperature and Ca (Table 2, Figure 4E,F). Moreover, an interaction effect of Ca and temperature was observed, with a greater increase in body size with the highest Ca at the lower temperature (Table 2, Figure 4F).

### 3.3. Reproductive Traits

#### 3.3.1. Reproduction Output

**Figure 5:**
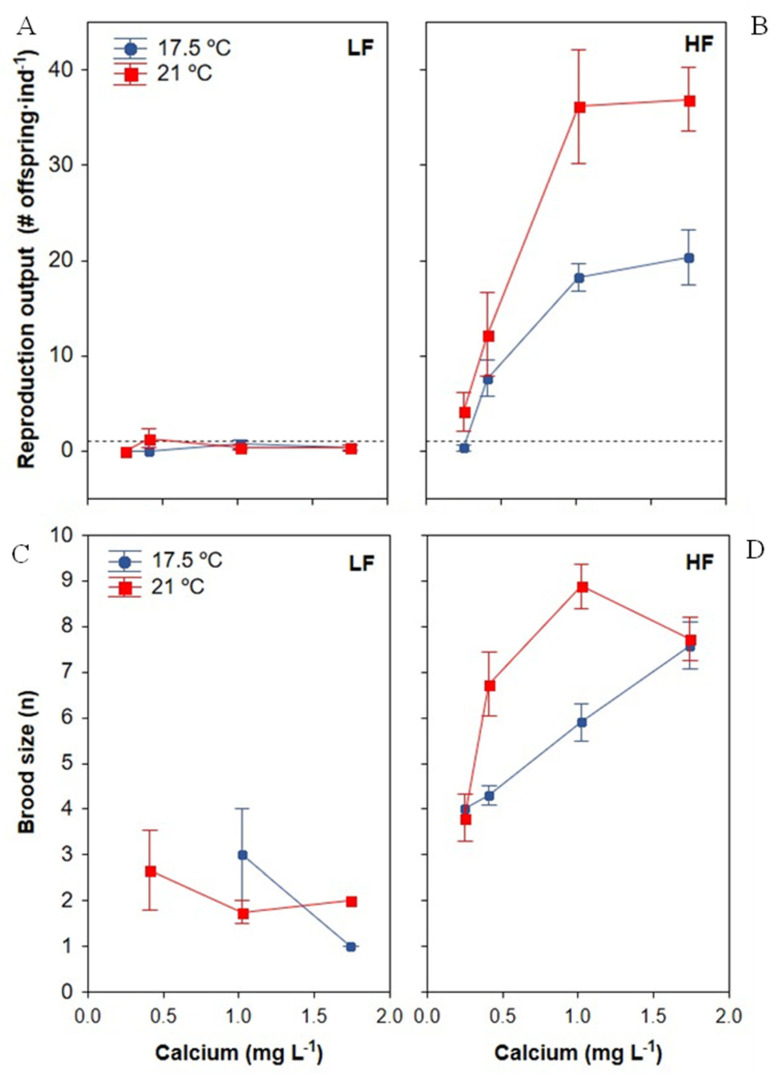
Effects of calcium concentration on reproductive traits of *D. pulex* at two temperatures and low (LF) or high (HF) food level. Data represent mean ± SE. (**A**,**B**): Reproduction output. The dotted line shows level reproduction output = 1 (i.e., each individual could give rise to one offspring but not itself survive, and the population size would stay approximately constant); (**C**,**D**): Brood size. Error whiskers are not given for the 21 °C LF 1.74 mg Ca L^−1^ and 17.5 °C HF 0.25 mg Ca L^−1^ treatments because only one experimental female produced offspring.

As expected, a higher food concentration enhanced the reproduction output of daphniids (Table 3, Figure 5A,B). No significant effect of Ca or temperature was observed on offspring production. At HF, however, *Daphnia* increased offspring production with higher Ca (Figure 5A,B; Ca × food interaction term, Table 3) at both temperatures. At HF, the minimum Ca concentration required for the *D. pulex* clone to produce offspring differed between temperatures (Figure 5B), being 0.25–0.41 mg Ca L^−1^ at 17.5 °C, whereas 33% of individuals reproduced at least once within the experimental timeframe with only 0.25 mg Ca L^−1^ when the temperature was 21 °C. The Ca reproduction threshold for the *D. pulex* clone was therefore <0.25 mg Ca L^−1^ at 21 °C. In contrast, the reproduction output at LF was <1 or slightly above 1 at all Ca and temperature levels (Figure 5A).

Results also showed a significant impact of the combined effect of increasing food and temperature (Table 3), with temperature having a greater effect on reproduction at HF than at LF (Figure 5A,B). The non-significant Ca × temperature interaction term was removed from the final model. However, the most intriguing result was the significance of the three-interaction term (Table 3), suggesting a complex interaction of the three predictors on the total number of *Daphnia* offspring; in this way, the higher temperature increased the effect of Ca on *Daphnia* offspring production when food was non-limiting (Figure 5B). Finally, no difference in reproduction output was observed above 1 mg Ca L^−1^ at either temperature (Figure 5B), suggesting a saturation point for reproduction at 1 mg Ca L^−1^, regardless of the temperature.

#### 3.3.2. Brood Size

The fertility (average number of neonates in each clutch) was significantly affected by food and temperature, while the effect of the Ca gradient depended on the food level (interaction *p* < 0.05, Table 2). *Daphnia* fertility was significantly greater when exposed to higher food concentration, and it was also increased by higher temperature but only at intermediate Ca concentration when food was non-limiting (Figure 5C,D). Higher Ca increased fertility at HF but not at LF, with fertility remaining between 1 and 4 offspring across the Ca gradient at LF but increasing from 3.85 ± 0.40 (SE) to 7.67 ± 0.35 (SE) with 0.25 and 1.74 mg Ca L^−1^, respectively, at HF. The largest broods (8.88 ± 0.49 (SE) neonates) were produced with 1.02 mg Ca L^−1^ at HF and 21 °C.

### 3.4. Population Growth Rate

**Figure 6:**
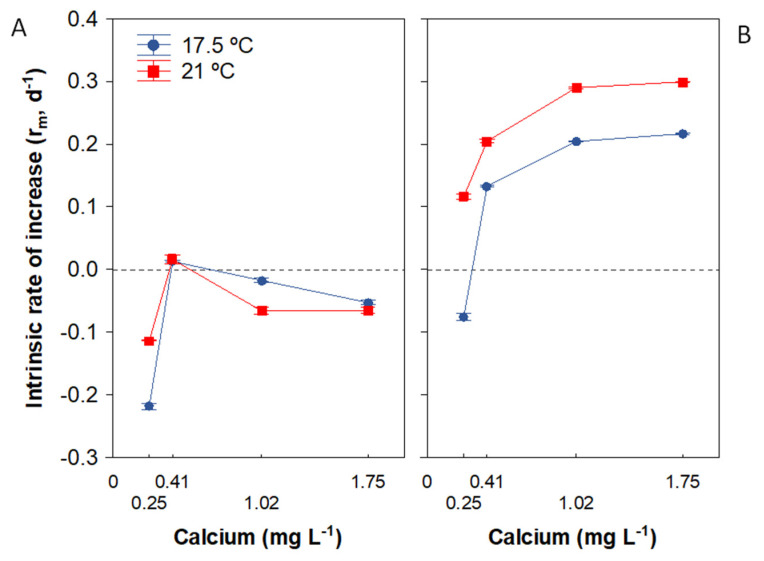
Average population growth rate (*r*) of *Daphnia pulex* in response to a Ca gradient at two temperatures with low (**A**) or high (**B**) food levels. Data represent mean ± SE. Note that error bars are smaller than data points in some cases.

The LMM with permutations revealed that *Daphnia* population growth rate was significantly influenced by the food concentration and Ca and by their interaction (Table 3). At LF, *r* increased slightly with the Ca gradient, displaying negative mean *r* values for all Ca treatments except 0.41 mg Ca L^−1^. However, the Ca effect was stronger at HF, observing an increase in *r* with higher Ca (Figure 6). All *r* values were positive at HF except at 0.25 mg Ca L^−1^ and 17.5 ℃, indicating that the Ca_ZPG_ at this temperature was between 0.25 and 0.41 mg Ca L^−1^ (Figure 6B), being 0.27 ± 0.00 (SE) mg Ca L^−1^ with a 95% confidence interval of 0.2735–0.2759. All animals had positive *r* values at 21 °C. The Ca_ZPG_ estimate for *Daphnia* was marginally significant at 21 °C (*p* = 0.069), being 0.17 ± 0.02 (SE) mg Ca L^−1^ with a 95% confidence interval that crosses zero (−0.07–0.41). This result suggests that Ca_ZPG_ varied between temperatures, as also reflected by the LMM, in which the interaction of Ca and temperature on *r* was significant (Table 3).

A summary of the most relevant outcomes of this study is given in Table 4.

## 4. Discussion

### 4.1. Food and Temperature Influence on the Calcium Threshold for Survival

No daphniids survived at 0.25 mg Ca L^−1^ for 19 d (Figure 3A–D); hence, the Ca survival threshold for the *D. pulex* clone was higher than 0.25 mg Ca L^−1^ regardless of food level or temperature. This value falls within the threshold range for *Daphnia* species (0.1–0.5 mg Ca L^−1^) proposed by other authors [7,11], although this range can be narrowed to 0.25–0.41 mg Ca L^−1^ for *D. pulex* at 17.5 °C. The temperature would not be expected to influence this Ca threshold range according to Ashforth & Yan [7]; at HF, however, the higher temperature raised the survival threshold of the *D. pulex* clone to 0.41–1.02 mg Ca L^−1^. This is the first study to show that the Ca survival threshold for *D. pulex* is influenced by temperature.

Importantly, the present findings indicate that the survival of *Daphnia* populations could be seriously reduced by rising temperatures, regardless of the availability of food and water Ca. Moreover, in the present survival model, independently of the experimental temperature, the effect of Ca was more pronounced under HF versus LF conditions (Ca × food interaction term, Table 1; Figure 3A–D). Similar results were reported by Pérez-Fuentetaja & Goodberry [9] for a hybrid *Daphnia pulex × pulicaria* raised at 20 °C. According to the present findings, when the increase in temperature is within their preferred range, the survival of *Daphnia* is primarily constrained by the energy (carbon) supplied by food despite high Ca, as also proposed by Ashforth & Yan [7], when the increase in temperature is above the preferred range (up to 28 °C).

### 4.2. Food and Temperature Influence on the Calcium Threshold for Reproduction

*D. pulex* development and reproduction were highly sensitive to the constraints in food quantity and Ca and the change of temperature. A striking observation on reproduction traits was the complex interaction of the three tested predictors on reproduction output (Table 3), revealing that the effect of Ca on *Daphnia* reproduction largely depended on the food quantity but that this effect varied according to the growth temperature. A positive synergistic effect of Ca and temperature on *D. pulex* reproduction was found when food quantity was non-limiting (Figure 4B). The total number of offspring, age of maturity and average brood size at 21 °C were comparable to those documented in other laboratory experiments with high food conditions at 20 °C for *D. pulex-pulicaria* (see e.g., [21]). However, in contrast to previous results [7,9,11], offspring were absent or extremely low at LF-0.2 mg C L^−1^ (Figure 4A), although adults surviving for more than 19 d would be expected to have reproductive capacity. The lack of reproduction or [Ca] effect under the present LF conditions (0.2 mg C L^−1^) suggest that this food level was below or very close to the threshold carbon level for the reproduction of this *D. pulex* clone (Figure 4A).

Under HF conditions, the *D. pulex* clone produced offspring at ≥0.25 mg Ca L^−1^ at both temperatures, (Figure 4D,F and Figure 5B), and no difference in reproduction output was observed above 1 mg Ca L^−1^ (Figure 5B). Hence, the reproductive threshold Ca for the *D. pulex* clone at around 20 °C was <0.25 mg Ca L^−1^ and the saturation point for reproduction was 1 mg Ca L^−1^, regardless of the temperature. This Ca threshold is within the Ca reproduction threshold range proposed by earlier studies (0.1–0.5 mg Ca L^−1^) for *D. pulex* [7] and other *Daphnia* species [11,79]. The reproductive threshold Ca at 21 °C was lower than that at 17.5 °C under HF conditions, suggesting that a higher temperature improved tolerance to low Ca for *Daphnia* reproduction (Figure 5B). This result contrasts with the previous study by Ashforth & Yan [7], who observed no change in the reproductive threshold of their *D. pulex* clone under HF when the temperature increased from 20 to 28 °C. Although it is widely accepted that water Ca is the predominant source of Ca for *Daphnia* [80,81,82], it has been suggested that the combination of increased daphniid ingestion rates with the presence of Ca in algal food might restore the Ca balance in Ca-deficient water [83], although this proposition is not supported by definitive empirical evidence. In the present experiment, algae grew under non-limiting Ca nutrient conditions. Moreover, the larger body size of *Daphnia* observed at HF and 21 °C when [Ca] was <1.5 mg Ca L^−1^ (Figure 4F) could be associated with an increased filtering rate [33,84]. In this way, if daphniids can extract an additional amount of Ca from food, a higher filtering capacity at HF might have supplemented the low ambient levels of Ca in the present experiment, according to the hypothesis of Muyssen et al. [83].

Under LF conditions, reproductive maturity was severely delayed (Table 2, Figure 4A) and the fertility (Table 2, Figure 5C) and total number of offspring (Table 3, Figure 5A) were all reduced, regardless of Ca. Thus, the reproductive threshold Ca could not be estimated for LF treatments.

### 4.3. Food and Temperature Influence on the Calcium Threshold for Population Growth

Population growth rate is ultimately a matter of survival and reproduction. In this study, earlier age at maturity and increased brood size were observed in HF treatments (Table 2), and these two life history traits were major determinants of the *Daphnia* population growth rate. Negative *r* values or values around zero in all LF treatments, regardless of the Ca concentration, resulted from high mortality and low reproduction under conditions of severe energy limitation (0.2 mg C L^−1^). Although some individuals reached reproductive maturity (Figure 4A), they died before leaving offspring. In contrast, *r* was positive at HF at all Ca concentrations except for 0.25 mg Ca L^−1^ at 17.5 °C (Figure 6B). Interestingly, the tolerance of poor Ca differed between temperatures at HF, with a markedly lower Ca_ZPG_ value at 21 °C (Ca_ZPG_ < 0.25 mg L) than at 17.5 °C (Figure 6B), as also observed for reproduction output. Moreover, the Ca_ZPG_ at 21 °C was even lower than the Ca survival threshold for the *D. pulex* clone (0.25 mg Ca L^−1^). These results suggest a high allocation of energy to reproduction when the temperature reaches around 20 °C, allowing the population to succeed at such a low Ca concentration, although the survival rate is reduced. In contrast, Ashforth and Yan [7] described a negative effect of temperature increases from 24 to 28 °C on *Daphnia* population growth rate at HF across a wide Ca range (0.5–10 mg Ca L^−1^). They concluded that *D. pulex* populations are more susceptible to low Ca when the temperature increases and food concentration is low; however, the temperatures studied were above the preferred range for reproduction. Although the thermal optimum of the present *D. pulex* clone was not established, it could not be sustained over the long term in the laboratory at 25 °C. Hence, the two experimental clones might have different thermal optima. Moreover, the present clone was isolated from high-mountain lakes that have never exceeded 21 °C during the thaw, to the best of our knowledge. Therefore, we hypothesise that the combined effect of Ca deficiency and increased temperature on the population growth rate of *Daphnia* may differ depending on whether the warming is within their preferred temperature range or not. We propose that, under high food availability, if the increased temperature is within this range, as in our study, *Daphnia* populations increase tolerance to Ca limitation, because the higher temperature accelerates moulting and stimulates their growth and reproduction rates. However, when the increased temperature rises above this range, it is possible that *Daphnia* populations decrease their tolerance to Ca limitation, as observed by Ashforth & Yan [7]. Higher tolerance to low Ca could be particularly important for the persistence of *D. pulex* populations in many cold and soft-water lakes worldwide affected by climate warming and the resulting increase in algal biomass from enhanced nutrient concentrations (e.g., [85]). The present results also support the hypothesis of Jiménez et al. [52] of an increase in abundance of *D. pulex-pulicaria* in alpine lakes in Sierra Nevada (Spain) over the past century due to warming and greater atmospheric Ca input.

## 5. Conclusions

This study demonstrates the combined effects of low Ca concentration (<2 mg Ca L^−1^) and a 3.5 °C increase in temperature on the survival and reproduction of North American *D. pulex* raised within their optimum physiological temperature range at low and high food levels. Significant interactive effects were observed on the biological fitness components of this widely distributed *Daphnia* lineage, which might predict the interaction of water Ca and food quantity in a global warming scenario. Our results suggest that in many cold and Ca-limited lakes (e.g., alpine Mediterranean lakes and lakes with boreal climatic characteristics), the ongoing warming process and higher food availability could make their populations more tolerant of low Ca levels and increase population growth rates. This would alter the community structure of lake zooplankton and induce potential cascading effects on the food web. Nevertheless, the extrapolation of these results to actual field conditions should be made with caution and is subject to verification by accumulated field data.

## Figures and Tables

**Table 1:** Results of the discrete-time survival model with mixed effects to test the effects of calcium concentration, food concentration, and temperature on the hazard of mortality of *Daphnia*.

Effects	Estimate	SE	Wald z
Intercept	−5.422	1.750	−3.098 **
Calcium (Ca)	−0.237	0.228	−1.038 ^ns^
Food	0.144	0.183	0.784 ^ns^
Temperature	0.191	0.089	2.140 *
Interval	−0.017	0.017	−1.018 ^ns^
Ca × food	−1.058	0.267	−3.969 ***

^ns^ *p* > 0.05; * *p* < 0.05; ** *p* < 0.01; *** *p* < 0.001.

**Table 2:** Results of the GLMMs performed on development and reproductive traits to test the effects of calcium concentration (log-transformed data), food concentration, and temperature on the age of maturity (log-transformed), moulting rate of juveniles, body size, and average brood size of *Daphnia*.

	Age of Maturity			Molting Rate			Body Size			Brood Size		
Effects	Estimate	SE	Wald z	Estimate	SE	Wald z	Estimate	SE	Wald z	Estimate	SE	Wald z
Intercept	5.47	0.40	13.73 ***	−0.46	0.33	−1.40 ^ns^	999.5	215.2	4.64 ***	−10.39	3.50	−2.97 **
Calcium (Ca)	−2.07	0.54	−3.84 ***	0.04	0.01	3.56 ***	1172.4	395.0	2.97 **	−0.50	0.92	−0.59 ^ns^
Food	−0.36	0.02	−14.62 ***	−0.33	0.19	−1.72 ^ns^	239.7	24.1	8.86 ***	2.65	0.27	9.85 ***
Temperature	−0.15	0.02	−7.24 ***	0.05	0.02	2.77 **	38.6	11.9	3.24 **	0.63	0.18	3.45 ***
Ca × food	-	-	-	-	-	-	-	-	-	1.19	0.49	2.43 *
Ca × temperature	0.11	0.03	3.73 ***	-	-	-	−54.9	21.1	−2.60 **	-	-	-
Food × temperature	-	-	-	0.02	0.01	2.23 *	-	-	-	-	-	-

*** *p* < 0.001; ** *p* < 0.01; * *p* < 0.05; ^ns^ *p* > 0.05.

**Table 3:** Results of linear mixed-effects models (LMMs) with null model permutation tests for the effect of calcium concentration (log-transformed data), food concentration, and temperature on the reproduction output and population growth rate of *D. pulex*.

	Reproduction Output			Population Growth Rate		
Effects	Chi-Square	df	*p*-Value from Permutation	Chi-Square	df	*p*-Value from Permutation
Calcium (Ca)	1.4974	1	0.22426	25.99	1	**0.00033**
Food	19.9	1	**0.00033**	18.458	1	**0.00033**
Temperature	0.2692	1	0.56548	2.0402	1	0.17727
Ca × food	8.3336	1	**0.00533**	54.956	1	**0.00033**
Ca × temperature	-	-	-	23.914	1	**0.00033**
Food × temperature	28.78	1	**0.00033**	35.359	1	**0.00033**
Ca × food × temperature	13.37	1	**0.00100**	-	-	-

Values that are statistically significant are indicated in bold.

**Table 4:** Summary of combined effects of water Ca, food quantity, and temperature on life-history traits and population fitness of *D. pulex*.

Life-History Traits	Relevant Outcomes from This Study
Survival	Ca survival threshold ≥0.25 mg Ca L^−1^ (19 d endpoint); at HF, higher Ca survival threshold when temperature increases from 17.5 to 21 °C Survival greatly reduced by temperature rising from 17.5 to 21 °C, regardless of the availability of food and water Ca Ca × food: Higher hazard of mortality by Ca stress at HF
Development traits	
Age of maturity	Ca × temperature: Earlier with increasing Ca at lower temperature (17.5 °C); no effect of Ca gradient at higher temperature (21 °C)
Moulting frequency of juveniles	Greater when Ca increased Food × temperature: increased at higher temperature and HF
Body size	Ca × temperature: Greater increase in body size with highest Ca at lowest temperature; at Ca < 1.5 mg Ca L^−1^, interestingly, *Daphnia* was larger in warmer conditions
Reproduction traits	
Reproduction output	Ca reproduction threshold < 0.25 mg Ca L^−1^ (at 21 °C) At HF, Ca saturation point for reproduction at 1 mg Ca L^−1^ Ca × food: At HF, increased when Ca increased; at LF, higher Ca had no effect Food × temperature: Greater effect of temperature increases at HF than at LF Ca × food × temperature: Positive synergistic effect of Ca and temperature at HF; At HF, a higher temperature improved Ca tolerance with a lower minimum Ca required for reproduction output > 1 offspring·ind^−1^
Fertility (brood size)	Ca × food: Increased at higher Ca at HF, but not at LF
Population growth rate	Ca × food: Ca effect stronger at HF Ca × temperature: Ca threshold for population growth (Ca_ZPG_) varied between temperatures under HF; Ca_ZPG_ markedly lower for 21 ° *versus* 17.5 °C

LF: low food (0.2 mg C L^−1^); HF: high food (2 mg C L^−1^).

## Data Availability

Not applicable.
